# Increasing frailty is associated with higher prevalence and reduced recognition of delirium in older hospitalised inpatients: results of a multi-centre study

**DOI:** 10.1007/s41999-022-00737-y

**Published:** 2023-01-25

**Authors:** Geriatric Medicine Research Collaborative, Waleed Faheem, Taran Nandra, Sarah Richardson, David Saliu, Daisy Wilson, Thomas A. Jackson, Laura Magill, Lauren McCluskey, Rita Perry, Carly Welch, Daisy Wilson, Claire Copeland, Emma L. Cunningham, Daniel Davis, Jonathan Treml, Thomas Pinkney, Terrence Quinn, Peter Nightingale, Benjamin Jelley, Victoria Gaunt, Mary Ni Lochlainn, Kumudhini Giridharan, Mustafa Alsahab, Stephen Makin, Kelli Torsney, Jane Masoli, Lindsay Ronan, Jenni K. Burton, Oliver Todd, Joanne Taylor, Ruth Willott, Natalie Cox, Roisin Healy, Nedaa Haddad, Sharan Ramakrishna, Zahid Subhan, Antonella Mazzoleni, Olga Nynaes, Jodie Crofts, Emily McNicholas, Hannah J. Robinson, Thyn Thyn, Jonathan Baillie, William McKeown, Caroline Rice, Gerrard W. Sloan, Katherine Williamson, Yasmeen Hayat, Vee Han Lim, Katie Millichamp, Amr Bazaraa, Angharad Chilton, Alexander Harbinson, John Headlam, Elisabeth Hunter, Zainab Hussain, Al Wakkass Mahmood, Liji Ng, Srividya Sundara, Felicia Tan, Alice Wheeler, Sophie Wright, Jack B. G. Baldwin, Kate O’shea, Ghazal Hodhody, Kara Mayor, Riana Patel, Shiv Bhakta, Marie Goujon, Adriana Jakupaj, James Dove, Matthew Kearney, Vincent McCormack, Kirsty Moore, Leo Pope, Hussun-Ara Shah, Megan E. Shaw, Gemma M. Smith, Ryan Love, Maya Mukundan, Muhammad Shahid, Ahmad Alareed, Karen Beharry, Ganapathy Bhat, Sanojan Bremakumar, Laurence Caines, Sandra Darko, Nisha Rai, Pedro Vila De Mucha, Phillipa Adams, Helen McDonald, Sam Mills, Frances Parry, Frances Rickard, Stuart Winearls, Rinata Farah, Robert Grange, Fiona Herbert, Elizabeth Lonsdale-Eccles, Qurrat Ul Ain, Hannah Watson, Celine Bultynck, Chiara Cavaliere, Cal Doherty, Sarah Evans, Daniel Furmedge, Annabel Hentall MacCuish, Esther Hindley, Caitlin Meyer, Emma Mullarkey, Rosamund Pullen, Aidan Ryan, Dhruv Sarma, Elaine Seymour, Katharine Stambollouian, Darmiga Thayabaran, Chenxian Wu, George Peck, Mahrukh Raza, Kapil Sahnan, Amber Dhillon, Oluwatosin O. Abiola, Catherine Bryant, Rachael Bygate, John Frewen, Isabel Greaves, Olivia Morrow, Simon Tetlow, Guy Tinson, Aayenah Yunus, Simran Bedi, Olivia Evans, Leeying Giet, Abolfazl Behbahani, Saurav Bhattacharya, Clare Hunt, Rajeev Mishra, Louise Connor, Jack Poynton, Elizabeth Deacon, Rory Durcan, Emma Fisken, Susan A. Hall, Jane Noble, Emma L. Prendergast, Ajay Macharouthu, Victoria Macrae, Emily Murphy-Ackroyd, Emily C. Rose, Nicola Watt, Mairi Blair, Eilidh R. Mackenzie, Roisin McCormack, Sophie J. Irwin, Alice Einarsson, Ming Khor, Baraa Alhadadi, Ewen Cameron, Angela Campbell, Eileen Capek, Lorna Christie, Laura Connell, Alan Cook, Jordanna Deosaran, Marianne Elliott, Elizabeth Evans, Sarah Fancourt, Sarah Henderson, Ambreen Hussain, Karen Jones, Dominic Kelly, Catherine M. McErlean, Hazel Miller, Fariha Naeem, Caroline Ostrowski, Megan Parkinson, Fadi Sami, Alison Shepherd, Lindsay Whyte, Emily Wright, Eilidh E. C. Ferguson, Michael Gallagher, Heather J. McCluskey, Louise Beveridge, Hollie A. Clements, Jasmine Hart, Neil Henderson, Su Kwan Lim, James E. Lucocq, Alison McCulloch, Adam A. Murray, Esther E. Y. Ngan, Philippa K. Traill, Amy Walter, Michael S. J. Wilson, Abigail Wrathall, Zhi Jiun Yap, Clementine Anderson, Hashem Abu-Arafeh, Ilan Gluck, Oliver Mitchell, Richard Robson, Elizabeth L. Sampson, Arunkumar Annamalai, Jamal Bhatti, Laura Briggs, Debbie Fraser, Jonathan Gui, Eleanor Leah, Natasha R. Matthews, Pryankaran Mithrakumar, Mohammad Moad, Michael Sen, Jacqui Straughan, Roxana Taranu, Kasim Usmani, Ayesha Aamir, Amaka Achara, Olugbenro Akintade, Elizabeth J. Ellis, Sneha Gurung, Chioma Iwu, Abdullah B. Khalid, Sejlo Koshedo, Shonit Nagumantry, Nader Nashed, Philip Nwabufor, Ijeoma T. Obi, Parrthiepan Visvaratnam, Edward Wu, Marc Bertagne, Peter Jackson, James Allen, Harriet Brown, Jennifer Champion, Natasha Christodoulides, Olivia Handley, Fiona E. Macdonald, Laura J. Beeley, Victoria Clayton, Aaron Kay, John Marshall, Hannah Morgan, George Naish, Sarah L. Cleaver, Jenny Evans, Abbie Morrow, Raj Amarnani, Khai L. Cheah, Claire Cushen, Amy Enfield-Bance, Martin Glasser, Suriti Govindji, Shama Mani, Jemma Gregory, Puja Jatti, Asma Khan, Helena Lee, Helen Millner, Huma Naqvi, Emily Williamson, Teresa Harkin, Bushra Khizar, Anna Lewis, Hannah Pendleton, Steve Rutter, Rohan Ahmed, Farah Coffey, Beth Hackett, Elizabeth A. M. Holmes, Ali Khan, Zeeshan Mustafa, Mark A. Bowman, James Irvine, Katherine Patterson, Sarah Horner, Ting W. Wong, Christopher Cairns, Kate Foster, Alex Hornsby, Robbie Horton, Laura Jones, Rachel King, Emily Lyon, Ani Tencheva, Faye Wilson, Lesley J. Young, Sindhoora Dama, Eleanor Giblin, Lleika Kunaselan, Olivia Lowes, Reema Menezes, Abigail Taylor, Alex Timperley, Rachel Batho, Charlotte Bell, Sammy Carter, Paul Croft, Eliza Griffiths, Giles Hall, William Hunt, Holly Jacques, Felicity Leishman, Seema Murthy, Sinead Quinn, James Reidm, Amy Walker, Stephanie A. Matthews, Ayoub Behbahani, Martin Glasser, Ana Silva Ferreira, Caroline Ashton, Swetha Byravan, Laura Cummings, Sana Faruq, Sarah Jagdeo, Philip Thomas, Karen Broadhurst, Joseph B. Wilson, Helen Bowden, Katrin Hoffman, Howell T. Jones, Charles Katz-Summercorn, Ethan Khambay, Lucy Porter, James Speed, Keziah Austin, Farrah Bahsoon, Rose Laud, Jawad Ali, Niall Fergusson, Claire Wilkes, Laura Babb, James Gaywood, Jessica Green, Ada Kantczak, Katy F. Madden, Sasha Porter-Bent, Moe Su Su San, Laxmi Babar, Helen Chamberlain, Tamsin Cricklow, Alexis Giles, Abhishek Gupta, Clare Hughes, Tammy Lee, Anum Cheema, Yathu Matheswaran, Asiodu Nneamaka, Anekea Ross, Tarunya Vedutla, Theresa J. Allain, Emily Bowen, Julie Dovey, Natalie Gaskell, Deborah Scott, Emma Stratton, Miriam Thake, Stuart Bullock, Siobhan McKay, Stephanie Radoja, Sherif Abdelbadiee, Samuel Cohen, Jane Giddings, Christopher J. Miller, Emma Mumtaz, Minal D. Patel, Vishnu Prasad, Lahiru Satharasinghe, Mark Studley, Marylin Browne, Sabrina K. Durrant, Emma Jay, Alex McQuillan, Megan Offer, Jessel Varghese, Alexander Baron, Christian Chourot, Peter Jackson, Kimberley Kirrane, Helen Rayner, Kate Tantam, Ebrahiem Tumi, Shabnam Venkat, Nisha Aggarwal, Emma Astaire, Karthik Basker, L. Berwick, Edward Bilton, Aimee-Louise Chamboult, George Chapman, Jasmine Chevolleau, Grace Fenneley, Shannelle House, Nathan Ingamells, Emilia Jewell, Vickneswaran Kalyaani, Mahum Kiani, Nagarjun Konda, Anusha Kumar, Jo Lai, Jamie Large, Joanna Livesey, Zeinab Majid, Jack McCready, Hannah Moorey, Bethan Morgan, Kirty Morrison, Alice Mosley, Adam Pailing, Sophie Pettler, Shayan Rashid, Lucy Rimmer, Danielle Scarlett, Gurpreet Sehmbi, Abigail Smith, Nina Smith, Charles Sprosen, Emily Taylor, Jemima Taylor, Connie Tse, Sophie Turton, Henry Vardon, Jasmine Virk, Sarah Warwicker, Hannah Woodman, Beth L. Woodward, Luke Wynne, Ollie Yule, Asim Ahmad, Paapa Appiah-Odame, Ciaran Barlow, Dorothy K. C. Kuek, Isabelle Nicholls, Emma Norman, Wioletta Pyc, Ashish Vasudev, Lawrence A. T. Adams, Emma Box, Chung Sien Chai, Darcy S. Wilson, Bogna Drozdowska, Emma Elliott, Adam Stirling, Martin Taylor-Rowan, Hannah Webb, Li Wong, Ahmed Abras, Muhammad Adam, Zarah Amin, Olivia Cooper, Rhianna Davies, Wan Idoracaera C. Ikhwan, Georgia R. Layton, Awolkhier Mohammedseid-Nurhussien, Sohail Shakeel, Hana Waraich, Jabed Ahmed, Katie Ball, Kwasi Debrah, Valerie Page, Zhao Xiao Bei, Hannah McCauley, David McShane, Freya Cooper, Natalie Grundmann, Michael Haley, Andre Le Poideven, Sarah B. McClelland, Emily Moore, Norman Pang, Hannah Currie, Jayne Davies-Morris, Sarah Edwards, Sureena Janagal, Rodric Jenkin, Polly Jones, Gary Kumbun, Sarah Parry, Bhavyas Tyagi, Janine Valentine, Saad Abdullah, Emmy Abu, Sarah Ahmad, Bilquis Ahmed, Hamza Ahmed, Ana Andrusca, Matthew Ansell, Thomas Arkle, Imola Bargaoanu, Charlotte Chuter, Katie Houldershaw, Jacqueline Ibanichuka, Shoaib Iqbal, Angela Kabia, Ismail Kadir, Anjli Krishan, Adam McClean, Gerry McGonigal, Pranav Mishra, Gladys Ofoche, Anna Reay, Simon M. Stapley, Adam Swietoslawski, Nihaad Syed, Karthika Velusamy, Afnan Wahballa, James Wilcockson

**Affiliations:** Institute of Inflammation and Ageing, University of Birmingham, Birmingham, B152TT UK

**Keywords:** Frailty, Delirium, Mortality, Recognition

## Abstract

**Purpose:**

Delirium is a neuropsychiatric disorder delineated by an acute change in cognition, attention, and consciousness. It is common, particularly in older adults, but poorly recognised. Frailty is the accumulation of deficits conferring an increased risk of adverse outcomes. We set out to determine how severity of frailty, as measured using the CFS, affected delirium rates, and recognition in hospitalised older people in the United Kingdom.

**Methods:**

Adults over 65 years were included in an observational multi-centre audit across UK hospitals, two prospective rounds, and one retrospective note review. Clinical Frailty Scale (CFS), delirium status, and 30-day outcomes were recorded.

**Results:**

The overall prevalence of delirium was 16.3% (483). Patients with delirium were more frail than patients without delirium (median CFS 6 vs 4). The risk of delirium was greater with increasing frailty [OR 2.9 (1.8–4.6) in CFS 4 vs 1–3; OR 12.4 (6.2–24.5) in CFS 8 vs 1–3]. Higher CFS was associated with reduced recognition of delirium (OR of 0.7 (0.3–1.9) in CFS 4 compared to 0.2 (0.1–0.7) in CFS 8). These risks were both independent of age and dementia.

**Conclusion:**

We have demonstrated an incremental increase in risk of delirium with increasing frailty. This has important clinical implications, suggesting that frailty may provide a more nuanced measure of vulnerability to delirium and poor outcomes. However, the most frail patients are least likely to have their delirium diagnosed and there is a significant lack of research into the underlying pathophysiology of both of these common geriatric syndromes.

**Supplementary Information:**

The online version contains supplementary material available at 10.1007/s41999-022-00737-y.

## Key summary points

**Aim:**

Describe frailty in hospital inpatients with delirium.

**Findings:**

Patients with delirium were more frail than patients without delirium. Higher frailty, as defined by CFS, was associated with reduced recognition of delirium.

**Message:**

Careful screening for delirium in frail older adults is essential in hospital inpatients.

**Supplementary Information:**

The online version contains supplementary material available at 10.1007/s41999-022-00737-y.

## Introduction

Delirium is an acute, fluctuating confusional state, which develops over hours to days [1]. It causes disturbances in attention, consciousness, and cognitive function and is associated with considerable distress along with poor outcomes including mortality, institutionalisation, and cognitive decline [2, 3]. Recognising delirium is crucial for clinicians from all specialties, with overall point prevalence in adult inpatients of 19.6% [4]. However, delirium is often not diagnosed in the clinical setting [5, 6].

Frailty is a state in which an individual has a reduction in their physiological capacity to respond to external stressors [7]. The Clinical Frailty Scale (CFS) is a nine point scale (ranging from 1—very fit, to 8—very severely frail, with 9—representing the terminally ill who are not otherwise frail) which is scored based on clinical judgement following a thorough history from the patient of their level of function 2 weeks prior to admission [8]. A number of methods for measuring frailty exist, but the CFS is rapid and relatively straightforward to complete, and has been shown previously to correlate highly with the longer Frailty Index (*r* = 0.8) [8]. Increasing frailty, described with the CFS, has been shown to correlate with worsening of outcomes including mortality rates, readmission to hospital, and longer hospital stays in emergency surgical inpatients, independent of age [9].

Previous work has shown that frailty increases the risk of delirium and increases the risk of mortality from delirium [10, 11]. However, there is a significant paucity of data regarding the relationship between frailty severity and delirium risk and outcomes, with the few studies that do exist shown to be highly heterogenous [10]. For the first time, we aimed to explore how frailty throughout the spectrum of severity, as measured using the CFS, affected delirium rates and recognition in hospitalised older people in the United Kingdom.

## Methods

### Study design and setting

We completed a multi-centre study of delirium screening and recognition in three rounds at acute hospitals across the United Kingdom. The results of rounds 1 and 2 have been published previously [12, 13]. Participation was open to all acute hospitals and the data were collected by local staff who volunteered; no financial incentives were provided. Following registration guidelines, data collection proforma and educational tools were shared via online resources. Round 1 was carried out prospectively on 14th March 2018, coinciding with World Delirium Day. Round 2 was a retrospective study on 14th September 2018 with data collected from the patient notes. Round 3 occurred on 13th March 2019 (World Delirium Day) acting as a full re-audit of Round 1 and used the same methodology. Collected data from round 1 were anonymised and entered into pre-formatted excel sheets and then collated in a central database. REDCap, a secure encrypted data collection software, was used for subsequent rounds.

### Participants

Inclusion criteria for round 1 were all patients aged ≥ 65 years admitted as an emergency to any specialty during the 48-h period preceding data collection on 14th March 2018 and who were still inpatients at the time of assessment. Round 2 included all patients aged ≥ 65 years admitted as an emergency to any specialty between 00:00 and 23:59 on 14th September 2018. Round 3 inclusion criteria were all patients aged ≥ 65 years admitted as an emergency to any specialty within the 24-h period which was 48–72 h prior to 8 am on 13th March 2019.

Exclusion criteria for all rounds were: patients admitted to critical care or those imminently dying, elective admissions, patients with clinical records which were unobtainable, or any other logistical reasons. Patients without complete data for frailty were excluded in this analysis.

### Delirium screening and assessment

For Rounds 1 and 3, collaborators at each site assessed patients between 08:00 and 20:00 on World Delirium Day 2018 and 2019. Patients were assessed using 4AT and those who scored ≥ 4 had further assessment by a clinician using DSM-5 criteria. Patients were then classified as definite delirium (all DSM-5 criteria met), possible delirium (some DSM-5 criteria met), or no delirium. Additional data were collected from the patient notes including age, gender, dementia status, and specialty, along with whether delirium screening was completed and delirium status documented. Clinical Frailty Scale (CFS) was recorded prospectively following review of the notes and a clinical assessment.

Round 2 was a retrospective analysis. The notes of all patients identified by the inclusion and exclusion criteria were analysed using a method of retrospectively diagnosing delirium from the notes that has been validated [13, 14]. The data were recorded as above.

Data were collected by individuals with training and understanding of delirium and frailty. The data were collected on structured proformas and collated using a structured database. All individuals collecting data were supervised by an individual with specialist training in geriatric medicine.

Unrecognised delirium is delirium, identified by the assessors using DSM-5 criteria, which has not been identified and/or recorded in the patient’s notes by the parent team.

### Statistical methods

Statistical analysis was performed using IBM SPSS Statistics 22 (Chicago, IL, USA). Differences between patients with and without delirium were analysed using chi-squared tests for categorical data and Mann–Whitney *U* test for continuous data. Possible delirium was coded as no delirium, and probable dementia was coded as dementia.

Binary logistic regression was performed to assess the effect of covariates upon delirium prevalence and recognition. Any missing variables and outcome data were coded as missing data, but these participants were included in all analysis, provided that data were available on the presence or absence of delirium and CFS.

### Ethical approval

All data were collected as part of a multi-centre audit to assess compliance with NICE guidelines and registered through clinical governance departments. Anonymised data were securely transferred to the University of Birmingham. Ethical approval was obtained for a secondary analysis of the anonymised database from the University of Birmingham Science, Technology, Engineering, and Mathematics Ethical Review Committee (ERN_18-1415A).

## Results

The study included 3013 unscheduled admissions over 82 UK hospital sites. Seventy-six patients were excluded from this analysis as data on frailty were missing. This included 1465 (49.9%) patients from round 1, 655 (22.3%) from round 2, and 817 (27.8%) from round 3. The characteristics of the study population for each round have been previously published elsewhere [15]. Delirium prevalence was 16.4% (483/2937). The patients with delirium were older, frailer, and more likely to have dementia (Table 1).

**Table 1:** Demographics of patients included within this study

	No delirium (*N* = 2454)	Delirium (*N* = 483)	*p* value
Gender—%female (*N*)	53.7% (1301)	58.5% (280)	0.055
Frailty—median CFS (IQR)	3 (1–4)	6 (5–7)	< 0.001
Age—median (IQR)	80 (73–86)	83 (78–89)	< 0.001
Dementia—% (*N*)	14.1% (346)	42.6% (205)	< 0.001
Screening—%screened (*N*)	28.0% (28)	43.9% (212)	< 0.001
Speciality			
Acute medicine	30.6% (751)	34.6% (167)	< 0.001
Geriatrics	17.6% (431)	34.8% (168)	
Stroke	4.2% (106)	1.4% (7)	
Other medicine	27.2% (686)	17.3% (85)	
Other surgery	4.6% (113)	0.8% (4)	
General surgery	7.7% (188)	3.9% (19)	
Orthopaedic surgery	7.8% (190)	7.0% (34)	
Length of stay—median (IQR)	7 (3–13)	10 (6–19)	< 0.001
Mortality—% (*N*)	4.9% (117)	13.5% (64)	< 0.001
Discharged to new care home—% (*N*)			
Institution	4.0% (36)	11.6% (17)	< 0.010

Table displays the demographic descriptive data of all patients included within this study. Gender, dementia, screening, speciality, death, and institutionalisation are displayed as proportion of total patients with missing data excluded. Chi-squared tests have been used to demonstrate statistical significance. Age, frailty, and length of stay are displayed as median with inter-quartile range. Data are not normally distributed. Mann–Whitney *U* tests have been used to demonstrate statistical significance

The data in the table describe the patients with delirium as older, frailer, and more likely to have dementia. The patients with delirium are more likely to die, be institutionalised, and have a longer length of stay

**Fig. 1:**
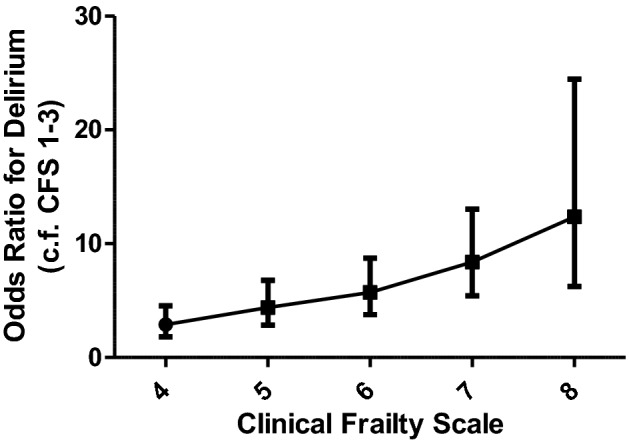
Risk of delirium with frailty. The odds ratio of prevalent delirium with increasing frailty measured by Clinical Frailty Scale. The figure demonstrates increasing risk of delirium with increasing frailty

The risk of delirium by CFS is shown in Fig. 1 and increases incrementally with increasing frailty score. Patients with a CFS of 4 were nearly three times more likely to develop delirium (OR [95% CI] 2.88 [1.83–4.55]) and patients with a CFS of 8 were 12 times more likely to develop delirium (OR [95% CI] 12.36 [6.24–24.46]) compared to patients considered not frail (CFS 1–3) (Supplementary material Table 1). This risk is independent of age, dementia, speciality, and gender.

**Fig. 2:**
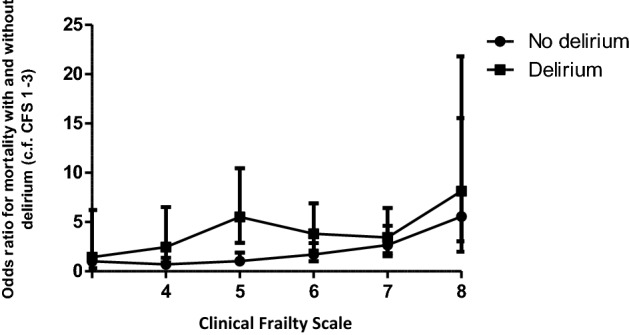
Mortality in patients with and without delirium. The odds ratio of mortality for patients with and without delirium plotted against frailty measured using the Clinical Frailty Scale. The figure demonstrates that mortality increases with increasing frailty and patients with delirium are more likely to die

Frailty and delirium were independent predictors of mortality, and therefore, mortality rate was highest in those with frailty and delirium (Fig. 2). Patients with a CFS of 8 with no delirium were five times more likely to die than patients with a CFS of 1–3 with no delirium (OR [95% CI] 5.55 [1.98–15.54]) and eight times more likely to die if they had delirium (OR [95% CI] 8.14 [3.04–21.80]). The effect of delirium on mortality was consistent throughout the CFS.

The effect of frailty on length of stay was significantly different for those with and without delirium (two-way analysis of variance of log, *p* = 0.013). Length of stay did not vary significantly with increasing frailty in individuals with delirium (one-way analysis of variance of log, *p* = 0.263). However, in individuals without delirium, length of stay increased with increasing frailty (one-way analysis of variance of log, *p* < 0.001 and *ρ* = 0.222, *p* < 0.001).

**Fig. 3:**
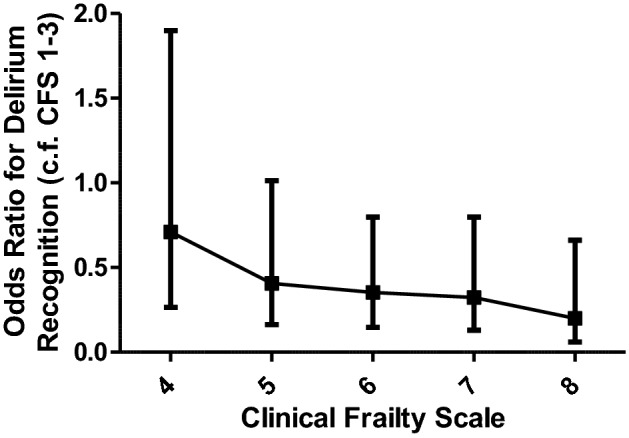
Recognition of delirium in frailty. The odds ratio of delirium being recognised by the clinical team plotted against frailty measured using the Clinical Frailty Scale. The figure demonstrates that recognition of delirium decreases with increasing frailty

When examining overall rates of delirium recognition, 48.9% (236/483) of cases of delirium were recognised. Delirium was less likely to be recognised by the clinical team in those who were more frail, with increasing CFS corresponding to a reduced likelihood of delirium being recognised compared to patients considered not frail (CFS 1–3) (CFS 1–3 vs CFS 8: OR 0.20) (Fig. 3 and Supplementary material Table 2). This risk is independent of age and sex.

## Discussion

For the first time, this large, multi-centre study has shown that there is an incremental increase in risk of delirium with increasing frailty according to the CFS. Importantly, this study also showed that the more frail a person is, the less likely it is that their delirium will be recognised by the clinical team caring for the patient and the worse their outcomes.

The relationship between delirium and frailty is not well understood, with few published studies combining the two common geriatric syndromes. A meta-analysis demonstrated that frailty is an independent risk factor for delirium, increasing the risk of subsequent delirium 2.2 times, but the studies included were highly heterogenous in terms of their study populations and the definitions used for frailty and delirium, making the comparison of studies challenging [10]. A further limitation of this and previous work was that frailty was defined as a dichotomous variable, not providing insight into the possibility of variable risk of delirium with different severities of frailty, as has been shown with mortality [9].

The CFS has become increasingly familiar to a wide variety of clinicians with its inclusion in mainstream guidelines [16]. We demonstrated previously that the CFS could be used at scale with minimal training [12]. Each additional point on the CFS has been shown to be associated with an increased risk of both mortality and institutionalisation [8, 9]. We have additionally shown that each additional point is associated with an increasing risk of delirium, with a CFS of 8 associated with four times the risk of delirium than a CFS of 4. We have also demonstrated that the addition of delirium to frailty increases the risk of mortality and this risk is consistent throughout the CFS. The increased mortality in frail patients with delirium has been previously demonstrated in an ICU population, but the relationship between frailty, mortality, and delirium has not been previously explored [17]. This suggests that CFS can be used to risk stratify patients for delirium, with those who are most frail highlighted as being at highest risk of delirium and the highest risk of death.

Length of stay in frailty was differently affected for patients with and without delirium. In patients with delirium, length of stay was not significantly different through the CFS, but in patients without delirium, length of stay increased with increasing frailty. This suggests that the effect of delirium nullifies the effect of frailty on length of stay. This interesting relationship has never been previously described and could be due to the complexities of discharge in patients with changes in cognitive health and the time required to recover from delirium.

In our study, we found that the frailer a patient, the less likely their delirium was to be recognised by their clinical team. This represents a significant concern and missed opportunity. Whilst we did not replicate these data in our recent study [13], other studies have demonstrated that outcomes are worse in those patients in whom delirium is missed [6]. The reasons for this lack of recognition of delirium in the most frail are likely to be complex, but may be due to a misperception by staff that more frail patients are expected to have cognitive impairment, and so, if present, this is often wrongly assumed to be chronic. Conversely, less frail patients may not be ‘expected’ to be confused, and so, the presence of confusion may trigger further screening and investigation. It is important for clinicians to recognise this cognitive bias and the negative impact this has on delirium diagnostic rates and outcomes in the most frail.

Interestingly, we found that the risk of unrecognised delirium in frail patients was independent of dementia. Although there is uncertainty regarding the diagnosis of delirium superimposed on dementia [18], our results show equal proportions of missed delirium in those with and without dementia. This is in line with previous work [5] and supports validation studies for the 4AT which demonstrated similar sensitivities, although slightly lower specificities, in people with and without dementia [19].

Frailty and delirium share many common features: both are multifactorial conditions and are associated with poor outcomes. It has been proposed that similar pathophysiology underlies both conditions [20], but the precise mechanism underlying the relationship between frailty and delirium is unknown. Delirium can be considered the manifestation of a final common pathway in multiple acute conditions. Key processes that lead to a vulnerability to delirium include changes in brain connectivity, neuroinflammatory, and vascular changes [21]. Frailty is also associated with a disruption to the immune function and a pro-inflammatory profile along with changes to metabolism and the vasculature [22, 23]. The processes that are associated with frailty also lead to a vulnerability to delirium.

The methodology used has several limitations, which have been described previously [12]. Relevant to this analysis, participants were assessed for delirium on just one occasion, which may have missed their delirium due to the fluctuating nature of the condition. This is likely to underestimate the true incidence of delirium. The retrospective ascertainment of data used in the second round of data may be biased by poor documentation and missing data. However, when we explored this further with our data, rates of prevalent delirium were similar. Illness severity was not recorded, and therefore, we were unable to adjust for this in our analysis. Major strengths of this project were the large number of participants and sites from throughout the United Kingdom and the recording of frailty as ordinal levels of severity.

## Conclusions

By recording frailty severity as an ordinal variable in our large, multi-centre cohort, we have shown that increasing CFS is associated with higher risk but lower recognition or delirium. This demonstrates that the CFS may be a useful tool for risk stratifying patients for delirium on admission to hospital and emphasises the importance of routine screening for delirium in all patients.

## Supplementary Information

Below is the link to the electronic supplementary material.

Supplementary file1 (DOCX 15 KB)

## Data Availability

The anonymised dataset is available from the corresponding author upon reasonable request.
