# Supplementary material for: Increasing frailty is associated with higher prevalence and reduced recognition of delirium in older hospitalised inpatients: results of a multi-centre study
Source: Eur Geriatr Med. 2023 Jan 25;14(2):325–32. doi: 10.1007/s41999-022-00737-y (PMC10113325; doi:10.1007/s41999-022-00737-y)
Supplement: Supplementary file 1 — Supplementary file1 (DOCX 15 KB) [file 41999_2022_737_MOESM1_ESM.docx]

**Supplementary Material**

**Table S1 – Logistic Regression of Co-variables for Risk of Delirium**

| **Co-Variables** | **Odd’s Ratio** | **Lower Confidence Interval** | **Higher Confidence Interval** |
| --- | --- | --- | --- |
| Age | **1.017** | 1.003 | 1.031 |
| Male | 0.877 | 0.704 | 1.093 |
| Geriatrics | 1.109 | 0.848 | 1.449 |
| Stroke | **0.475** | 0.313 | 0.721 |
| Other Medicine | **0.628** | 0.445 | 0.886 |
| Other Surgery | **0.252** | 0.090 | 0.710 |
| General Surgery | 0.734 | 0.431 | 1.248 |
| Orthopaedics | 0.833 | 0.534 | 1.299 |
| Dementia | **2.271** | 1.789 | 2.882 |
| CFS 4 | **2.880** | 1.825 | 4.546 |
| CFS 5 | **4.375** | 2.827 | 6.772 |
| CFS 6 | **5.724** | 3.750 | 8.738 |
| CFS 7 | **8.410** | 5.413 | 13.067 |
| CFS 8 | **12.358** | 6.244 | 24.456 |

**Table S2 – Logistic Regression of Co-variables for Recognition of Delirium**

| **Co-Variables** | **Odd’s Ratio** | **Lower Confidence Interval** | **Higher Confidence Interval** |
| --- | --- | --- | --- |
| Age | 1.014 | 0.987 | 1.043 |
| Male | 1.167 | 0.781 | 1.744 |
| Geriatrics | **1.713** | 1.072 | 2.737 |
| Stroke | **0.180** | 0.063 | 0.511 |
| Other Medicine | 1.047 | 0.551 | 1.988 |
| Other Surgery | 0.913 | 0.117 | 7.116 |
| General Surgery | **0.094** | 0.020 | 0.434 |
| Orthopaedics | 0.506 | 0.223 | 1.151 |
| Dementia | **1.845** | 1.201 | 2.834 |
| Hyperactive | 0.701 | 0.357 | 1.376 |
| Mixed | 0.590 | 0.281 | 1.236 |
| Hypoactive | **0.401** | 0.217 | 0.738 |
| CFS 4 | 0.710 | 0.265 | 1.900 |
| CFS 5 | 0.405 | 0.162 | 1.012 |
| CFS 6 | **0.352** | 0.146 | 0.853 |
| CFS 7 | **0.322** | 0.130 | 0.797 |
| CFS 8 | **0.199** | 0.060 | 0.660 |
